# Experimental and Modeling Investigations on the Water Sorption Behaviors of Autoclaved Aerated Concrete

**DOI:** 10.3390/ma14216235

**Published:** 2021-10-20

**Authors:** Halina Garbalińska, Magdalena Bochenek, Marcin Stasiak

**Affiliations:** 1 Faculty of Civil and Environmental Engineering, West Pomeranian University of Technology in Szczecin, Al. Piastów 50a, 70-311 Szczecin, Poland; Magdalena.Bochenek@zut.edu.pl; 2 Institute of Mathematics, Faculty of Automatic Control, Robotics and Electrical Engineering, Poznań University of Technology, Piotrowo 3A, 60-965 Poznań, Poland; Marcin.Stasiak@put.poznan.pl

**Keywords:** autoclaved aerated concrete, sorption isotherms, moisture, air humidity, modeling approaches

## Abstract

The thermal and moisture properties of building envelope materials determine their performance over many years of use. Moisture has a particularly negative impact, impairing all the technical parameters and adversely affecting the microclimatic conditions inside the building. This article presents research and analysis on the moisture behavior of partitions made of autoclaved aerated concrete. Autoclaved aerated concrete is a very popular material for building external walls because of its relatively good thermal insulation and sufficient strength, if it is not subjected to increased moisture. This study investigated how the moisture content of this material changes with the change in relative air humidity. The four most popular density classes were studied. The sorption isotherms were determined by the static gravimetric method throughout the whole hygroscopic range. Moreover, the applicability of various models to describe sorption isotherms of this material group has been extensively evaluated. The tested models (Peleg, Redlich, Chen, Oswin, Henderson, Lewicki, Caurie, and GAB) all provided a very good fit with the experimental results for the tested material group (R^2^ ranged from 0.9599 to 0.9978). This paper indicates that the use of two additional approximation parameters (SSE and RMSE) allows a more precise assessment of the quality of individual models.

## 1. Introduction

The properties of a building envelope made of hygroscopic materials strongly depend on the moisture content—with an increase in moisture, the thermal insulation performance of the partitions decreases (increased energy loss), and both the quality of the indoor climate (mold growth, increased VOC emissions) and the durability (physical, chemical, and biological damage) deteriorate. Therefore, significant attention has been paid to problems associated with the dampness of building materials, and in particular to the research and modeling of sorption isotherms.

Sorption isotherms describe the adsorption properties of a porous material. The shape of a sorption isotherm depends on the adsorbent microstructure (in particular, the pore size) as well as adsorbate–adsorbent interactions; therefore, different hygroscopic materials exhibit very different sorption isotherm characteristics.

**Figure 1:**
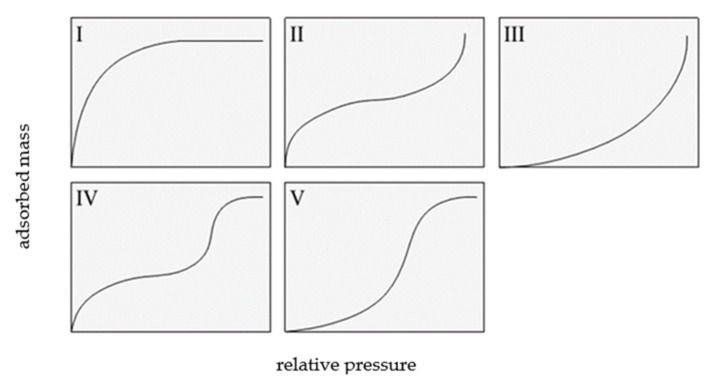
Types of isotherms according to Brunauer, based on [1].

Two classifications of sorption isotherms are most commonly cited in the literature: the Brunauer classification and the International Union of Pure and Applied Chemistry (IUPAC) [1,2]. The Brunauer classification includes five types of adsorption isotherms, which are shown in Figure 1.

**Figure 2:**
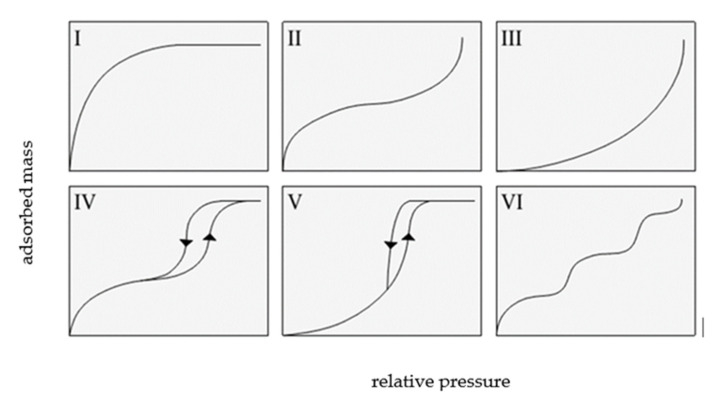
The types of isotherms according to IUPAC, based on [2].

The classification recommended by the International Union of Pure and Applied Chemistry (IUPAC) distinguishes six types of adsorption isotherms, as shown in Figure 2.

IUPAC isotherms of type I, II, and III overlap with type I, II, and III of the Brunauer classification.

A type I isotherm is produced by microporous adsorbents, where the micropores are rapidly filled at relatively low pressure. The shape of this isotherm is convex, and the amount of adsorbed gas slowly approaches a limit value as *p/p*_0_ increases. Type II isotherms display a characteristic inflection point, which indicates the moment of complete monolayer coverage and the onset of the formation of subsequent layers. Type III isotherms are produced by materials with very weak adsorbate–adsorbent interactions, and their shape is concave over the entire *p/p*_0_ range, without an inflection point.

Type IV and V isotherms exhibit a hysteresis loop and are produced by mesoporous materials in which capillary condensation occurs. At low relative pressures, a type IV isotherm corresponds to a type II isotherm, whereas a type V isotherm is characteristic of mesoporous materials, but with weak adsorbent interactions; thus, it shows some similarity with a type III isotherm.

A type VI isotherm describes multi-layer adsorption on a homogeneous surface. The steepness of the steps depends on the homogeneity of the adsorbent surface, the temperature, and the type of adsorbate [3,4,5].

Type I isotherms are best described by the Langmuir and Freundlich equations:

The Langmuir equation [3,6]:

(1) $$a=a_{m}\frac{Kp}{1+Kp}$$

where: *a*—the amount of adsorbed substance; *a_m_*—the amount of adsorbate corresponding to the coverage of the monolayer; *K*—the adsorption equilibrium constant; *p*—the adsorbate pressure.

The Freundlich equation [5,6]:

(2) a=kp1n

or,

(3) $$a=a_{m}{\left(Kp\right)}^{m}$$

where: *m*, *n*—empirically defined heterogeneity coefficients.

Type II–V isotherms are best described by the BET (Brunauer–Emmett–Teller) equation, which has the following form [3,4,6]:

(4) $$a=\frac{a_{m}c\frac{p}{p_{0}}}{\left(1-\frac{p}{p_{0}}\right)\left[1+\left(c-1\right)\frac{p}{p_{0}}\right]}$$

where: *p*/*p*_0_—relative partial pressure of water vapor; *p*_0_—saturated vapor pressure; *c*—constant.

The isothermal sorption of building materials (including aerated concretes) has been the subject of many research papers (e.g., [7,8,9,10,11,12,13,14,15]) that have focused on experimentally determining sorption isotherms, as well as their mathematical descriptions. In these studies, the influence of various factors on the equilibrium moisture level in a given material, e.g., autoclaved aerated concrete, was evaluated at different relative humidity levels.

**Figure 3:**
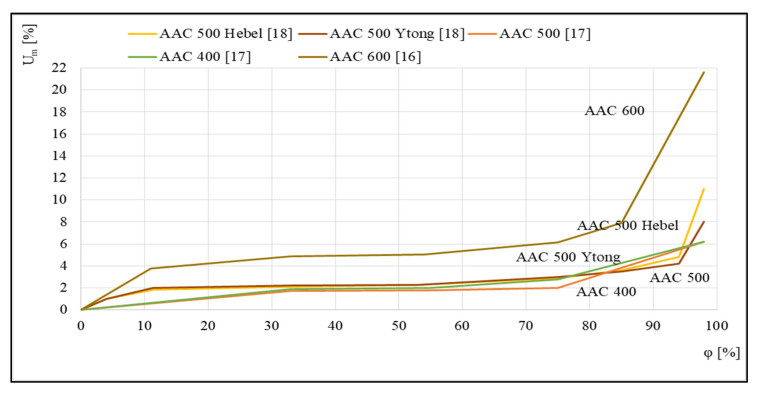
Sorption isotherms, based on [16,17,18].

Figure 3 shows examples of the sorption isotherms of several autoclaved aerated concretes that were studied in [16,17,18]. For the five different concretes presented, a relationship was discovered between the equilibrium mass humidity *U_m_* and the relative air humidity *φ*.

The runs illustrated in Figure 3 indicate a significant influence of aerated concrete density on the profile of the corresponding sorption isotherm. The intensification of the sorption process for concrete with the highest density of 600 kg/m^3^, which takes place over the entire tested humidity range *φ*, is worth noting.

**Figure 4:**
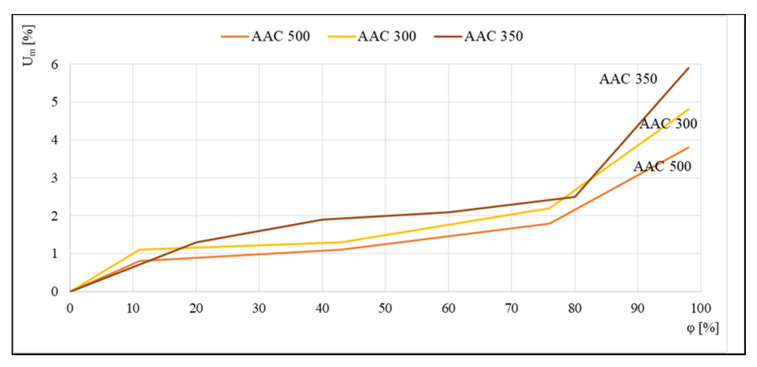
Sorption isotherms of class 300, 350, and 500 kg/m^3^ aerated concrete, based on [19].

The reverse effect is shown in Figure 4, where data were compiled to compare the sorption isotherms of aerated concrete of densities ranging from 300 to 500 kg/m^3^. Tests were carried out using the standard method in desiccators on 4 cm × 4 cm × 1 cm samples. Reproduced runs indicated that more aerated concretes had a higher sensitivity to moisture than class 500 concrete.

**Figure 5:**
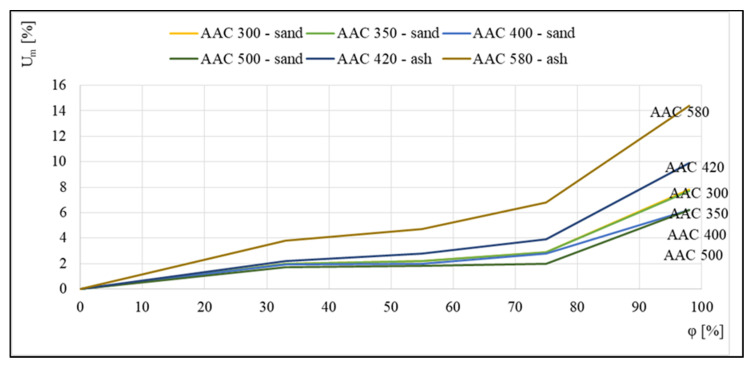
Sorption isotherms of 300, 350, 400, and 500 kg/m^3^ sand-based aerated concretes and 420 and 580 kg/m^3^ ash-based aerated concretes, based on [17].

Figure 5 illustrates the seemingly opposite trend, in which the highest equilibrium moisture values were noted for the concrete with the highest density of 580 kg/m^3^; however, this was related to the fact that this concrete, similar to 420 kg/m^3^ aerated concrete, is an ash concrete, which is characterized by higher sorption moisture contents relative to sand concretes.

The available data indicate that, in addition to density, the volume of adsorbed moisture was influenced by the composition of the raw materials as well as the production process. Non-autoclaved concretes, which are made of cement and fly ash, showed higher sorption capacities. The differences may also be related to the selection of autoclaving parameters.

It should be stressed that the acquisition of sorption isotherm data of porous building materials is very time-consuming because experiments must be performed under several conditions, such as different humidities *φ*, that must be stably maintained for many months. For this reason, many researchers have tried to find a mathematical model that can accurately predict the sorption isotherms of a given material. Among the most popular are the models proposed by Keller [3], Henry [5,6], Freundlich [5,6], Dubinin and Raduszkiewicz [4], Redlich-Peterson [20], Kisarow [21], Hüttig [22,23], Łykow [24], Hansen [25] Chen and Chen [26], Jovanovič [27], and many others; however, not all of them are suitable for describing the isotherms of building materials.

A comprehensive summary of the selected equations, together with the results of their adaptation to various types of building materials, can be found in [16]. It presents both experimental results and approximations of sorption isotherms for the most popular building materials: silicate brick, ceramic brick, aerated concrete, cement mortar, modified mortar, and cement-lime mortar. Tests on these materials were carried out at three environmental temperatures and at six levels of relative humidity.

This extensive dataset was then used in [16] to test 24 equations to assess their accuracy in describing the isotherms of each of the six analyzed building materials. In each case, the best match with the experimental data was found for the Chen function [26].

Taking into account the favorable results given in [16], the suitability of Chen’s model for describing the sorption isotherms of the four most popular density classes of aerated concrete (400, 500, 600, and 700) was also tested. In addition to Chen’s model, a few other models were selected that appeared promising in terms of their description’s accuracy, i.e., those that have since been applied in other areas, mainly the food industry.

## 2. Materials and Methods

Due to the complex interactions between various material and technological factors, the initial assumptions for the planned studies were specified beforehand. The purpose of those studies was to reconstruct the isotherms of aerated concretes that differed only in density and then to assess the accuracy of their model description using equations selected from the literature. Therefore, the purpose was to eliminate side factors that may have had an uncontrollable effect on the *U_m_* equilibrium values obtained at the different *φ* levels tested.

We decided to conduct experiments on the most common density classes: 400, 500, 600, and 700 kg/m^3^, which differ only in their pore structures. To eliminate the uncontrollable influence of other material and technological factors, we ensured that all test materials came from the same manufacturer, while guaranteeing that the same raw materials were used to produce concrete in each class, as well as the same technological parameters. Thus, sorption studies were performed on samples that differed only in density. The samples of each class served as four porous model mediums designed to recreate their isotherms and then evaluate the suitability of the tested models for describing them.

The raw materials used for AAC are sand, ground quicklime, cement, gypsum, alumina powder and water. Before sorption testing, composition tests of all autoclaved aerated concretes were performed. Table 1 summarizes the data for each AAC class tested.

**Figure 6:**
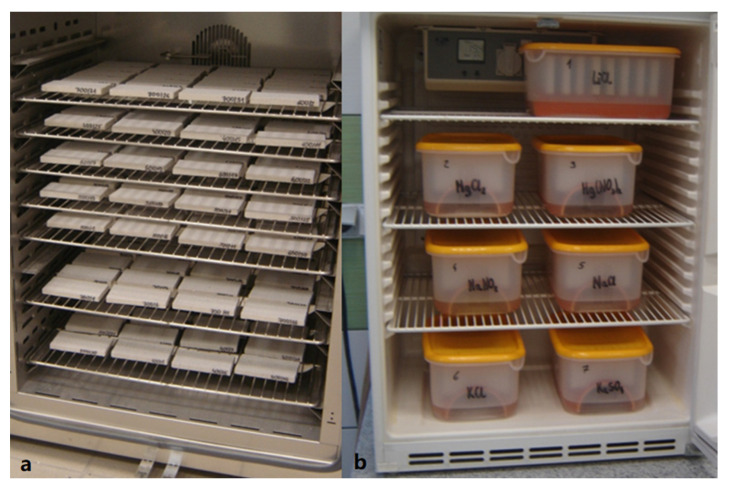
Samples dried to a constant mass, located in the dryer (**a**) and samples in closed containers placed inside the thermostatic chamber (**b**). Both photographs are from the authors’ collection.

Sorption studies were performed using the classical gravimetric method. For these tests, 4 perpendicular samples were taken from each class with a frontal surface area of 6 cm × 12 cm and a thickness of approx. 1 cm. These samples were cut from the internal part of the blocks for each density class. The actual sample density was 407 kg/m^3^ for AAC 400, 495 kg/m^3^ for AAC 500, 569 kg/m^3^ for AAC 600, and 665 kg/m^3^ for AAC 700, respectively. After the samples were surveyed, they were dried in a dryer to a constant weight at 105 °C, then insulated on their sides with a silicone coating and re-dried to a constant weight (Figure 6a). These prepared samples were placed on racks and sealed in containers with pre-prepared saturated aqueous solutions of appropriately selected salts at the bottom. These test systems were placed in two thermostatic chambers set to 20 °C, and each chamber contained 7 containers (Figure 6b).

The standard method of sorption testing of building materials (gravimetric static method) is described by European [28] and US standards [29], which provide two alternative methods for determining the sorption properties of porous building materials: using desiccators and using a climatic chamber.

In [30], both sorption and desorption processes were studied in autoclaved aerated concretes of four density classes (400, 500, 600 and 700). The testing was carried out by two independent methods, the traditional gravimetric Saturated Salt Solution (SSS) method and the Dynamic Vapor Sorption method (DVS). A comparison of sorption isotherms determined independently by each method (SSS and DVS) is presented in [31]. Analogous comparisons concerning desorption isotherms were presented in [32]. The research results provided in [31,32] show that the dynamic method (DVS) exhibits good agreement with the traditional method (SSS) over a wide range of relative humidity, excluding the highest humidity *φ* > 75%, at which the DVS method provides underestimates for both sorption isotherms and desorption isotherms.

This article presents the sorption results obtained using the standard method, which uses suitable hygrostatic solutions to stabilize the environmental humidity in each of the 7 containers. Solutions were prepared using the following salts: LiCl, MgCl_2_, Mg(NO_3_)_2_, NaNO_2_, NaCl, KCl, and K_2_SO_4_. Table 2 lists the relative air humidity *φ* over the saturated aqueous solutions of these salts at 20 °C.

The study of the sorption processes under 7 moisture humidity conditions lasted 10 months and was finished only when each sample reached equilibrium moisture content, allowing the *U_m_* value to be determined for all samples of each of the four classes tested.

In addition to these measurements, the suitability of several selected models for describing experimentally determined sorption isotherms of the four tested density classes of aerated concrete was evaluated.

Eight models in total were selected from the literature for further analysis. Chen’s model was evaluated first, which proved to be very accurate in [16]. It can be described in the form of an equation:

(5) $$U_{m}=\frac{a\varphi }{\left(1+b\varphi \right)\left(1-c\varphi \right)}$$

where: *U_m_*—mass moisture content; *a*, *b*, *c*—experimentally determined constants; *φ* = *p*/*p*_0_ relative air humidity; *p*—partial pressure of water vapor; *p*_0_—saturated vapor pressure.

In addition to Chen’s model, other models have been recommended in the literature to describe the moisture isotherms of autoclaved aerated concrete. The most commonly used models are the GAB model and the models proposed by Henderson, Oswin, and Lewicki [16,26]. These are described by Formulas (6)–(9), with the symbols represented by: *U_m_*—mass moisture content; *φ*—relative air humidity; *a*, *b*, *c*, *K*, *F*, *G*, *H*—constants used in the different models:

The GAB model:

(6) $$U_{m}=\frac{a_{m}cK\varphi }{\left(1-K\varphi \right)\left(1-K\varphi +cK\varphi \right)}$$

The Henderson model:

(7) $$U_{m}=a{\left(-\ln\left(1-\varphi \right)\right)}^{b}$$

The Oswin model:

(8) $$U_{m}=a{\left(\frac{\varphi }{\left(1-\varphi \right)}\right)}^{b}$$

The Lewicki model:

(9) $$U_{m}=F\left(\frac{1}{{\left(1-\varphi \right)}^{G}}-\frac{1}{1+\varphi^{H}}\right)$$

In this paper, we evaluated the accuracy of the above-mentioned five models recommended in the literature for describing the sorption isotherms of aerated concretes, as well as three additional ones. These models were selected in their own field after conducting preliminary analyses to assess their suitability. Their validity was demonstrated in other fields, mainly for modeling food sorption isotherms [35,36,37]. These were the models proposed by Peleg [38], Redlich-Peterson [20], and Caurie [39], by the following equations:

The Peleg model:

(10) $$U_{m}=k_{1}\varphi^{n_{1}}+k_{2}\varphi^{n_{2}}$$

The Redlich-Peterson model:

(11) $$U_{m}=\frac{K\varphi }{1+B\varphi^{n}}$$

The Caurie model:

(12) $$U_{m}=e^{A\varphi +B}$$

where: *U_m_*—mass moisture content; *φ*—relative air humidity; *A*, *B*, *K*, *n*, *k*_1_, *k*_2_, *n*_1_, *n*_2_—constants based on experimental tests (*k*_1_ > 1; *k*_2_ > 1; *n*_1_ > 1; 0 < *n*_2_ < 1 and 0 < *φ* < 1).

## 3. Results and Discussion

Table 3 shows the obtained U_m_ equilibrium values derived from sorption measurements carried out on four samples from each of the tested classes, with seven humidity levels arranged in the environment of the samples by the hygrostatic solutions described above. The data in Table 3 are shown on the top graphs placed in Figures 9–12. The shape of the measured isotherms corresponded to a type III isotherm in the Brunauer and IUPAC classifications. The sigmoidal shape of the water vapor sorption curves indicated the formation of multi-molecular water layers on the pore surfaces of the tested materials. There was no clear boundary in the transition from monolayer adsorption to multilayer adsorption. In the high-humidity range, capillary condensation was observed, which intensified the course of individual equilibrium curves to varying degrees.

The data included in Table 3 were collected during a PhD thesis [30]. These results correspond to equilibrium states, obtained after storing the samples for several months under particular moisture conditions. The results refer to a total of 112 cuboidal samples 1 cm × 6 cm × 12 cm, with 28 samples from each class.

Subsequently, an attempt was made to find the best fit of the theoretical curves to the experimental points.

Table 4 summarizes the individual parameters assigned to Equations (5)–(12) for the four AAC density classes tested, for all eight models.

Table 5 summarizes the calculated R^2^ (coefficient of determination), SSE (Sum of Squares Due to Error), and RMSE (Root Mean Squared Error) values to better assess the accuracy of each model. The SSE is the sum of the squares of the data errors—the closer the SSE value is to zero, the better the fit is. The RMSE can be used to indicate the accuracy of the model fit to the data—the lower the RMSE, the better the model fit.

By evaluating the values for each of the three parameters (R^2^, SSE, and RMSE), it was possible to more thoroughly diagnose the quality of the fit for each model.

**Figure 7:**
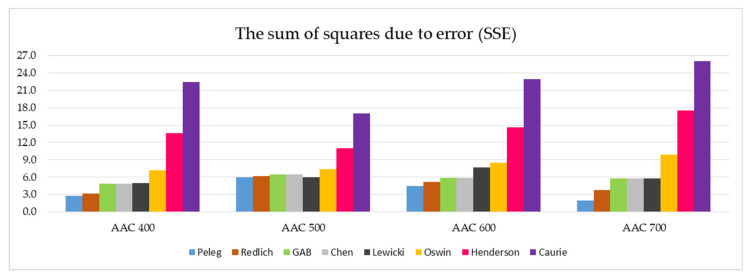
Graphical representation of the SSE values determined for all eight models analyzed and the four AAC density classes tested (data on individual values of SSE are presented in Table 5).

Figure 7 summarizes the SSE values of the eight models tested, which are divided into four AAC density classes.

**Figure 8:**
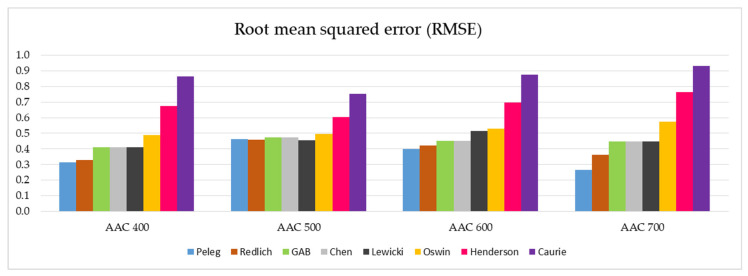
Graphical representation of the RMSE values determined for the eight models analyzed and the four AAC density classes tested (data on individual values of RMSE are presented in Table 5).

In a similar arrangement, the RMSE values of the tested models and the different AAC density classes are presented in Figure 8.

By analyzing the R^2^ values in Table 5, the most unfavorable values appeared for the AAC500 density class, and the R^2^ values in samples taken from this material ranged from ca. 0.986 (for the Peleg and Lewicki models) to ca. 0.960 (for the Caurie model). However, it should be noted that all R^2^ values indicated a very good accuracy of the fit for each of the tested models and for all density classes.

A more thorough assessment was possible by applying additional factors, i.e., SSE and RMSE.

The analysis of the SSE parameter values presented in Figure 7 reveals five models that are best suited to describe the sorption isotherms for all the tested autoclaved aerated concretes. This group includes the Peleg, Redlich, GAB, Chen, and Lewicki models. Apparently weaker in the evaluation are the other three models: Oswin, Henderson and Caurie. In particular, the last two stand out negatively in terms of the SSE coefficient value.

The analogical observations concerning individual models can be made after the analysis of the RMSE parameters illustrated in Figure 8. Additionally, in this case, a group of five best suited models can be identified (Peleg, Redlich, GAB, Chen, and Lewicki) with relatively low values of RMSE parameters. The other three models, in particular the Henderson and Caurie models, do not qualify for a positive evaluation.

In-depth analysis of the data summarized in Figure 7 and Figure 8 indicates that the Peleg model has the most favorable SSE and RMSE parameters.

**Figure 9:**
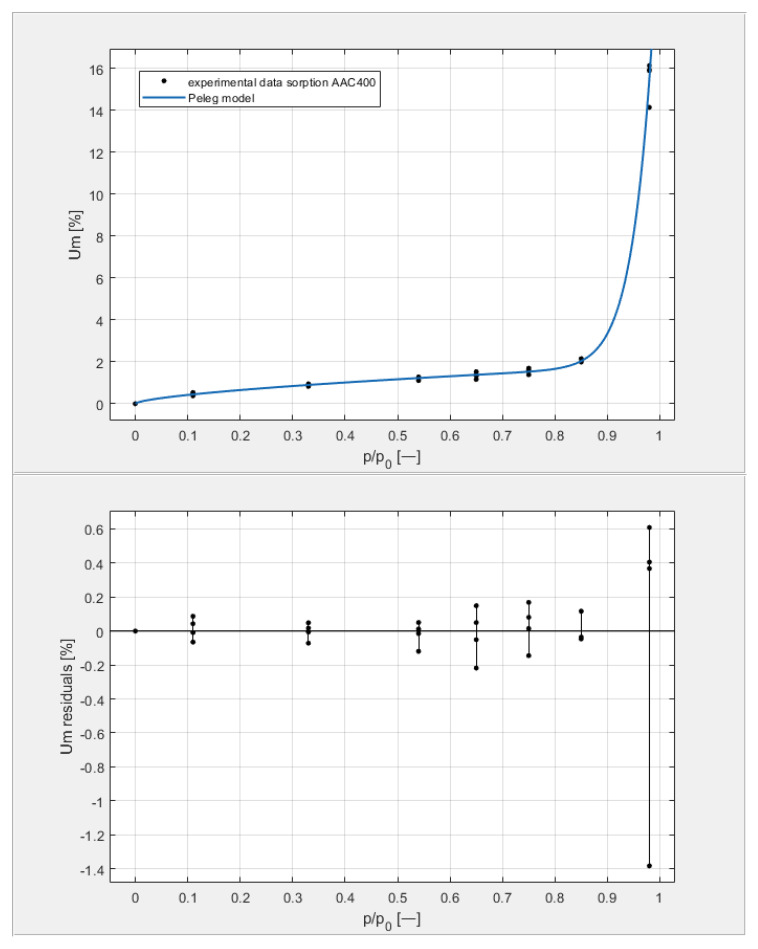
The sorption isotherms of class 400 aerated concrete described by the Peleg equation, plotted with the approximation error.

**Figure 10:**
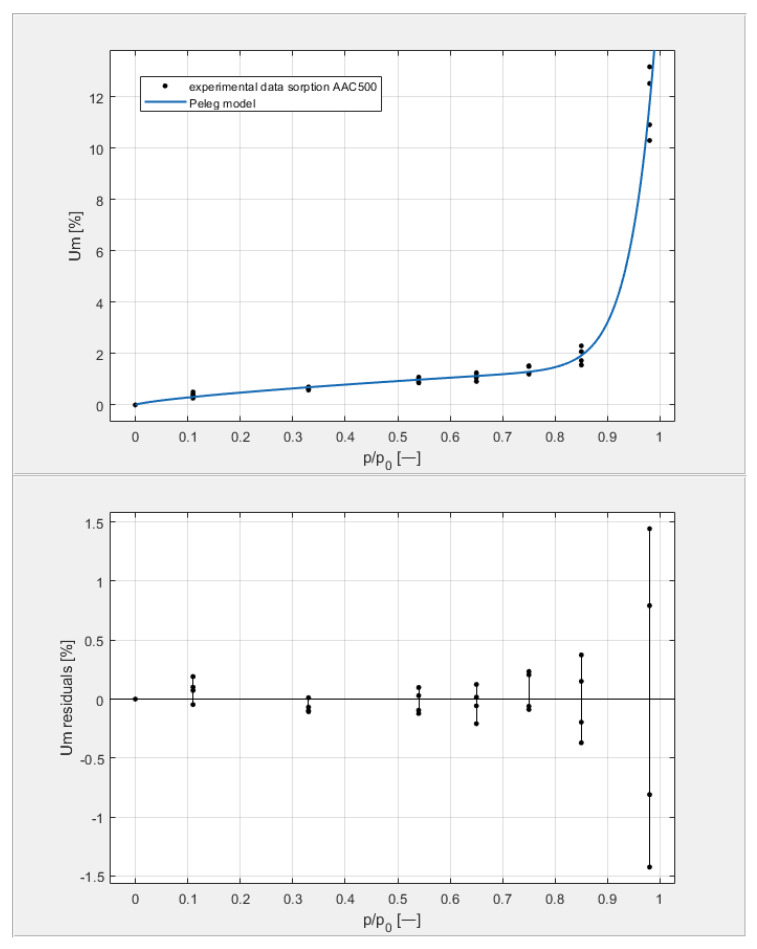
The sorption isotherms of class 500 aerated concrete described by the Peleg equation, plotted with the approximation error.

**Figure 11:**
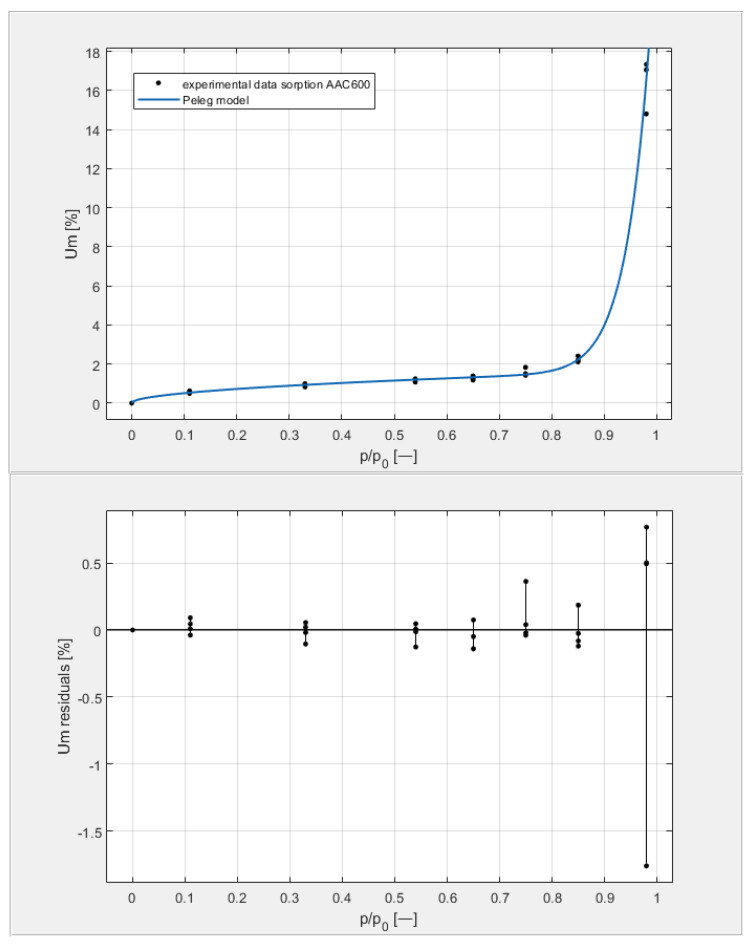
The sorption isotherms of class 600 aerated concrete described by the Peleg equation, plotted with the approximation error.

**Figure 12:**
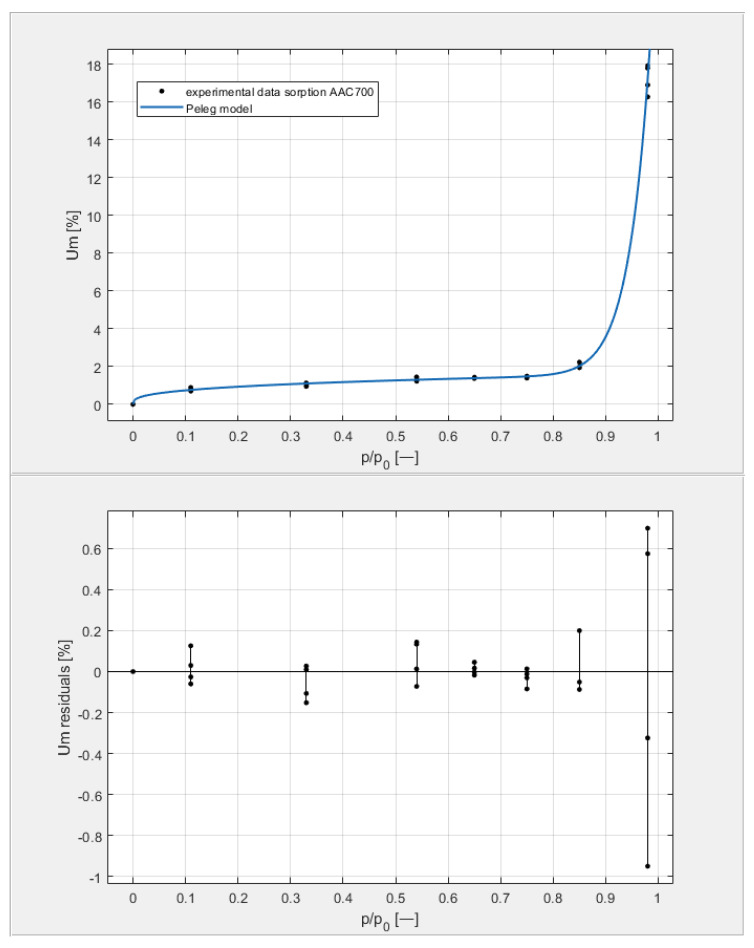
The sorption isotherms of class 700 aerated concrete described by the Peleg equation, plotted with the approximation error.

Figure 9, Figure 10, Figure 11 and Figure 12 show the experimental values of U_m_ for individual concrete samples of classes 400, 500, 600, and 700, as well as graphs of the best fit of the Peleg model. Error charts are provided for each approximation.

By analyzing individual datasets, it can be concluded that due to the resulting approximation errors for each concrete, they show minimum error values at humidities *φ* = 11, 33, 54, 65, 75, and 85% at levels not exceeding 0.2% for AAC 400, 0.5% for AAC 500, 0.4% for AAC 600, and 0.2% for AAC 700. Slightly higher approximation errors (but still relatively low) were observed for the highest moisture level *φ* = 98%. Their values did not exceed 1.4% for AAC 400, 1.5% for AAC 500, 1.7% for AAC 600, and 1.0% for AAC 700.

## 4. Conclusions

Based on moisture sorption studies, this process was found to proceed with similar intensities in four classes of tested aerated concretes. The results indicated that the moisture content of the samples increased significantly as the relative humidity increased. The isotherms showed a gradual progression at relative humidity values below 75–85%. In contrast, a significant increase in moisture content occurred in the humidity range of 75–85% to 98%.

The sorption isotherms of all studied aerated concretes were sigmoidal and belonged to type III isotherms, according to the Brunauer and IUPAC classifications.

It should be emphasized that experimental determination of the sorption isotherms of porous building materials is very labor- and time-consuming. Testing at different humidity levels needs to be arranged and then the changing mass of samples needs to be measured systematically over a period of many months in each climate. Despite this, scientists undertake this type of research because of the relevance of sorption isotherms in the estimation of other material parameters (e.g., heat transfer coefficient), values of which are strongly related to the moisture content in the material. Therefore, among other things, the accuracy of the sorption isotherms reconstruction and the quality of the description of their course are extremely important.

This paper evaluates the quality of eight models—five (Chen, Oswin, Henderson, Lewicki, and GAB) that are recommended in the literature for the description of autoclaved aerated concrete isotherms and an additional three (Peleg, Redlich, and Caurie) that have so far been used mainly in relation to the food industry.

To assess the suitability of the analyzed models, three approximation parameters were used (R^2^, SSE and RMSE).

The R^2^ parameter is a common quality measure for a model under consideration. It takes values from 0 to 1, and the higher the value, the better the fit provided by the model. If R^2^ meets inequalities 0.9 < R^2^ < 1, the fit is considered good.

Restricted to the basic R^2^ parameter, every model would receive a positive recommendation due to the satisfactory R^2^ value ranging from 0.9599 (Caurie) to 0.9978 (Peleg).

The inclusion of the additional parameters SSE and RMSE in this analysis made it possible to carry out a more precise assessment and to identify five models (Peleg, Redlich, GAB, Chen, and Lewicki) ensuring a good quality of the sorption isotherms that fit to the determined experimental data. The lower the value of both of these approximation parameters, the more precise the description provided by the model. The values of the SSE and RMSE parameters summarized in Table 5 and visualized in Figure 7 and Figure 8 allow the selection of five models (Peleg, Redlich, GAB, Chen, and Lewicki) that ensure the sorption isotherms fit well with the experimental data. For all tested concretes (AAC 400, AAC 500, AAC 600 and AAC 700), the parameters SSE and RMSE were the lowest in Peleg’s model.

Based on the analyses carried out, it can be concluded that the Peleg method most accurately correlated the equilibrium moisture content of *U_m_* of all tested concretes with the relative air humidity *φ* = *p*/*p*_0_ across the whole hygroscopic range. The maximum approximation error was slightly above 1.5% for all experimental data.

Based on the data obtained and the conducted analyses, it is reasonable to evaluate the quality of the model on the basis of three approximation parameters, supplementing the basic parameter R^2^ with two additional parameters SSE and RMSE. This makes it possible to select the model that provides the most accurate description for the type of material tested.

Of course, the specific microstructural properties of the material should be considered separately. Due to the influence of the mineralogical composition and the particular microstructure of a porous material during its sorption processes, the authors plan to relate these material parameters to the sorption isotherms assigned to the individual classes of autoclaved aerated concretes tested.

## Figures and Tables

**Table 1:** Percent phase contribution.

Mineral	AAC 400 [%]	AAC 500 [%]	AAC 600 [%]	AAC 700 [%]
Rutile	0.04	0.02	0.05	0.06
Basanite	3.46	3.05	5.58	0.74
Anhydrite	9.48	4.82	3.20	4.23
Calcite 2	4.73	5.50	2.03	1.66
Calcite 1	0.73	0.47	0.18	1.50
Tobermorite	29.02	28.35	29.99	35.42
Quartz	52.54	57.79	58.97	56.39

**Table 2:** The steady-state values of relative humidity over saturated aqueous solutions of selected salts, based on [28,29,33,34].

Chemical Formula	Name (IUPAC)	*T* [°C]	*φ* [%]
LiCl	Lithium chloride	20	11
MgCl_2_	Magnesium chloride	20	33
Mg(NO_3_)_2_	Magnesium nitrate (V)	20	54
NaNO_2_	Sodium (III) nitrate	20	65
NaCl	Sodium chloride	20	75
KCl	Potassium chloride	20	85
K_2_SO_4_	Potassium sulphate (VI)	20	98

**Table 3:** Stabilized sorption moisture content U_m_ of the autoclaved aerated concretes of classes 400, 500, 600, and 700 [30].

*φ* [%]	AAC 400	AAC 500	AAC 600	AAC 700
	*U_m_* [%]	*U_m_* [%]	*U_m_* [%]	*U_m_* [%]
11	0.378	0.382	0.54	0.886
	0.486	0.41	0.494	0.7
	0.53	0.262	0.623	0.734
	0.435	0.5	0.577	0.79
33	0.822	0.698	0.952	1.126
	0.91	0.588	0.912	0.992
	0.942	0.579	0.827	0.947
	0.888	0.618	0.987	1.108
54	1.209	1.01	1.244	1.224
	1.274	0.858	1.184	1.309
	1.104	0.886	1.205	1.44
	1.236	1.079	1.07	1.43
65	1.327	1.14	1.393	1.363
	1.527	1.067	1.177	1.377
	1.428	0.916	1.269	1.426
	1.16	1.249	1.179	1.397
75	1.696	1.522	1.505	1.388
	1.608	1.228	1.827	1.461
	1.542	1.2	1.443	1.486
	1.382	1.494	1.426	1.442
85	2.15	1.727	2.201	1.937
	1.988	2.298	2.145	1.973
	1.987	2.074	2.412	1.973
	1.998	1.553	2.106	2.224
98	15.894	13.157	14.804	17.788
	16.134	10.905	17.33	17.911
	15.932	10.291	17.058	16.262
	14.143	12.507	17.066	16.888

**Table 4:** Summary of the determined coefficients of the analyzed models for the tested aerated concretes.

	Model Parameter	AAC 400	AAC 500	AAC 600	AAC 700
Peleg	k1	22.7	15.41	22.99	25.37
	k2	1.813	1.534	1.639	1.594
	n1	24.87	20.39	21.33	23.98
	n2	0.6387	0.7261	0.5099	0.3354
Redlich	K	−1.212	−1.036	−1.1	−1.359
	B	2.183	1.738	2.114	2.265
	n	16.85	9.523	11.35	22.03
Chen	a	1.21 × 10^4^	1.54 × 10^5^	1.21 × 10^6^	1.50 × 10^6^
	b	3.20 × 10^4^	4.57 × 10^5^	3.13 × 10^6^	3.93 × 10^6^
	c	0.9956	0.991	0.9965	0.9978
Oswin	a	0.6346	0.5998	0.6343	0.6084
	b	0.8208	0.763	0.8377	0.8581
Henderson	a	0.6135	0.6201	0.6144	0.5539
	b	2.366	2.151	2.413	2.517
Lewicki	F	0.4394	−0.9319	5.941	−11.82
	G	0.9144	−3.341	0.9914	−0.1096
	H	−6.571	4.103	1.098	31.87
Caurie	A	2.93 × 10^−5^	11.76	13.56	14.5
	B	13.44	−9.073	−10.49	−11.39
GAB	a_m_	0.3773	0.3375	0.388	0.3811
	c	3.03 × 10^6^	4.75× 10^5^	3.27× 10^6^	4.16× 10^6^
	K	0.9956	0.991	0.9965	0.9978

**Table 5:** Summary of SSE, R^2^, and RMSE approximation parameters for the analyzed models and the tested aerated concretes.

		AAC 400	AAC 500	AAC 600	AAC 700
Peleg	SSE	2.772	6.015	4.464	1.992
	R^2^	0.9963	0.9858	0.9948	0.9978
	RMSE	0.3146	0.4635	0.3993	0.2667
Redlich	SSE	3.124	6.13	5.158	3.791
	R^2^	0.9958	0.9856	0.9935	0.9959
	RMSE	0.3282	0.4598	0.4218	0.3616
Chen	SSE	4.883	6.518	5.895	5.781
	R^2^	0.9934	0.9847	0.9931	0.9937
	RMSE	0.4104	0.4741	0.4509	0.4465
Oswin	SSE	7.155	7.377	8.465	9.884
	R^2^	0.9901	0.9826	0.9901	0.9892
	RMSE	0.4884	0.4959	0.5312	0.574
Henderson	SSE	13.64	11.01	14.59	17.47
	R^2^	0.9817	0.9741	0.9829	0.9809
	RMSE	0.6742	0.6057	0.6974	0.7632
Lewicki	SSE	4.933	6.003	7.719	5.796
	R^2^	0.9934	0.9859	0.991	0.9937
	RMSE	0.4124	0.455	0.5159	0.4471
Caurie	SSE	22.41	17.03	22.94	26.06
	R^2^	0.9699	0.9599	0.9731	0.9715
	RMSE	0.8644	0.7533	0.8744	0.932
GAB	SSE	4.883	6.518	5.895	5.781
	R^2^	0.9934	0.9847	0.9931	0.9937
	RMSE	0.4104	0.4741	0.4509	0.4465

## Data Availability

Data available in a publicly accessible repository.
